# Naodesheng decoction regulating vascular function via G-protein-coupled receptors: network analysis and experimental investigations

**DOI:** 10.3389/fphar.2024.1355169

**Published:** 2024-03-12

**Authors:** Shuhan Chen, Ziran Niu, Yanjia Shen, Wendan Lu, Jiaying Zhao, Huilin Yang, Minmin Guo, Li Zhang, Ruifang Zheng, Guanhua Du, Li Li

**Affiliations:** ^1^ Beijing Key Laboratory of Drug Targets Identification and Drug Screening, Institute of Materia Medica, Chinese Academy of Medical Sciences and Peking Union Medical College, Beijing, China; ^2^ Department of Pharmacy, Peking Union Medical College Hospital, Chinese Academy of Medical Sciences and Peking Union Medical College, Beijing, China; ^3^ Xinjiang Key Laboratory of Uygur Medicine, Xinjiang Institute of Materia Medica, Urumqi, Xinjiang, China

**Keywords:** Naodesheng decoction, network analysis, molecular docking, G-protein-coupled receptor, vascular function, material basis

## Abstract

**Introduction:** Ischemic stroke (IS) is a detrimental neurological disease with limited treatment options. Recanalization of blocked blood vessels and restoring blood supply to ischemic brain tissue are crucial for post-stroke rehabilitation. The decoction Naodesheng (NDS) composed of five Chinese botanical drugs, including *Panax notoginseng* (Burk.) F. H. Chen, *Ligusticum chuanxiong* Hort., *Carthamus tinctorius* L., *Pueraria lobata* (Willd.) Ohwi, and *Crataegus pinnatifida* Bge., is a blood-activating and stasis-removing herbal medicine commonly used for the clinical treatment of cerebrovascular diseases in China. However, the material basis of NDS on the effects of blood circulation improvement and vascular tone regulation remains unclear.

**Methods:** A database comprising 777 chemical metabolites of NDS was constructed. Then, the interactions between various herbal metabolites of NDS and five vascular tone modulation G-protein-coupled receptors (GPCRs), including 5-HT1AR, 5-HT1BR, β2-AR, AT1R, and ETBR, were assessed by molecular docking. Using network analysis and vasomotor experiment of the cerebral basilar artery, the potential material basis underlying the vascular regulatory effects of NDS was further explored.

**Results:** The Naodesheng Effective Component Group (NECG) was found to induce relaxation of rat basilar artery rings precontracted using Endothelin-1 (ET-1) and KCl *in vitro* in a dose-dependent manner. Several metabolites of NDS, including *C. tinctorius*, *C. pinnatifida*, and *P. notoginseng*, were found to be the main plant resources of metabolites with high docking scores. Furthermore, several metabolites in NDS, including formononetin-7-glucoside, hydroxybenzoyl-coumaric anhydride, methoxymecambridine, puerarol, and pyrethrin II, were found to target multiple vascular GPCRs. Metabolites with moderate-to-high binding energy were verified to have good rat basilar artery-relaxing effects, and the maximum artery relaxation effects of all three metabolites, namely, isorhamnetin, kaempferol, and daidzein, were found to exceed 90%. Moreover, metabolites of NDS were found to exert a synergistic effect by interacting with vascular GPCR targets, and these metabolites may contribute to the cerebrovascular regulatory function of NDS.

**Discussion:** The study reports that various metabolites of NDS contribute to its vascular tone regulating effects and demonstrates the multi-component and multi-target characteristics of NDS. Among them, metabolites with moderate-to-high binding scores in NDS may play an important role in regulating vascular function.

## 1 Introduction

Stroke endangers human health due to its high morbidity, high disability, and high mortality and has become the second leading cause of death in the world. Ischemic stroke (IS), as the main type, accounts for 87% of the incidence of stroke (Ajoolabady et al., 2021). Cerebral ischemia causes a pathophysiological cascade reaction, and insufficient blood flow was poorly equipped to satisfy the requirement of oxygen for the cerebral tissue (Qin et al., 2022). Drugs that dilate the cerebral vessels can restore the blood supply and provide a material basis for post-stroke rehabilitation. Thrombolytic therapy is currently the main strategy for the treatment of ischemic stroke; recombinant tissue plasminogen activator (r-tPA) is the only FDA-approved drug for IS treatment in the United States, but due to its narrow treatment time window (<4.5 h) and side effects such as hemorrhagic transformation, only few patients can benefit from it (Powers et al., 2019). Therefore, it is imperative to develop new and effective drugs against cerebral ischemia.

Naodesheng (NDS) is an extensively used traditional Chinese medicine (TCM) for the treatment of cerebrovascular diseases in China (Zhang et al., 2009). This formula is composed of five Chinese botanical drugs, including 78 g of San Qi (*Panax notoginseng* (Burk.) F. H. Chen) (Araliaceae, *Notoginseng radix et rhizoma*), 78 g of Chuan Xiong (*Ligusticum chuanxiong* Hort.) (Umbelliferae, *Chuanxiong rhizoma*), 91 g of Hong Hua (*Carthamus tinctorius* L.) (Asteraceae, *Carthami flos*), 261 g of Ge Gen (*Pueraria lobata* (Willd.) Ohwi) (Fabaceae, *Pueraria*e *radix*), and 157 g of Shan Zha (*Crataegus pinnatifida* Bge.) (Rosaceae, *Crataegi fructus*). Modern pharmacological studies have demonstrated that NDS has the neural protective effects including alleviating the formation of free radicals, improving cerebrovascular disturbance, and diminishing new thrombi forming from cerebral injury induced by cerebral ischemia and reperfusion (Zhang et al., 2002; Cai et al., 2008; Chen et al., 2008). In addition, NDS was shown to reverse memory loss and cognitive function in Alzheimer’s disease (AD) by regulating Aβ metabolism, synaptic plasticity, and neuroinflammation (Yuan et al., 2014; Pang et al., 2018). Moreover, preclinical studies from our laboratory have illustrated that middle dose (0.02 g/kg) and high dose (0.07 g/kg) of the Naodesheng effective component group (NECG), the 30% and 60% ethanol fraction mixture of NDS, significantly extenuate the neurological deficit in rats 7 days after focal cerebral ischemic injury when compared with the ischemia/reperfusion (I/R) group. Moreover, the efficacy of the NECG is better than that of NDS (1.075 g/kg) (Zhang et al., 2009). NDS has been shown to improve blood viscosity and erythrocyte deformability in 4VO rats with cerebral ischemia (Cai et al., 2008), but the material basis of NDS in the treatment of stroke still remains unclear.

G-protein-coupled receptors (GPCRs) are the largest family of membrane receptors with seven transmembrane α-helix structures in the human body (Weis and Kobilka, 2018). In the state of disease, ligands bind to GPCRs and connect to multiple signal cascades to mediate vasoconstriction (Maguire and Davenport, 2005). Among them, the receptors related to regulating vasomotor are mainly 5-hydroxytryptamine receptor (5-HTR), β2-adrenergic receptor (β2-AR), angiotensin II type-1 receptor (AT1R), and endothelin B receptor (ETBR) (Stenman and Edvinsson, 2004; Steven et al., 2018; Islam et al., 2020; Islam et al., 2021; Ma et al., 2021). Cerebral vasodilation and contractile activity are tightly related to ischemic stroke. Mounting evidence implicates the participation of both GPCRs and their ligands, including 5-HT, Ang II, and ETB, in the modulation of cerebral ischemia (Justin et al., 2018; Aguiar et al., 2021; Gulati et al., 2021). Previous studies have demonstrated that NDS can suppress vascular restenosis by regulating vasomotor-related factors and can also ameliorate brain microcirculation in rats with blood stasis (Guo and Yang, 2009). However, the basic mechanism and interaction between the metabolites of NDS and vascular GPCRs require further elucidation.

Traditional Chinese medicine (TCM) has been practiced in China for thousands of years and has gained wide clinical application. In view of the complex composition of TCM, it has multi-target intervention effects, coupled with a variety of extraction and separation methods, which are time consuming. Therefore, the concept of network pharmacology was proposed by Hopkins (2008). Li (2007) introduced the research of bioinformatics and TCM compounds and further analyzed the association and mechanism of action between TCM compounds and diseases, laying a foundation for the research of TCM and network analysis. With an increasingly large number of target protein structures being solved, the molecular docking has been proved to be one of the most widely applied approaches and achieved a great success. It predicts the ligand–target interaction at the molecular level or describes the structure–activity relationship (SAR), usually by predicting the molecular orientation of ligands while interacting with recipients, then using the scoring function to estimate its complementarity and delivering a new network simulation method to explore the potential targets of drugs and improve the accuracy of prediction by the utilization of its multi-directional characteristics (Crampon et al., 2022). Moreover, molecular docking can be used to further verify and support the results of network analysis. However, up to date, the material basis of NDS in prevention of ischemic stroke has not been elucidated fully. The study offers a considerable possibility that, through molecular docking and network pharmacology analysis, the potential material basis of NDS can be further explored, which can help develop clinical strategies for IS.

**FIGURE 1:**
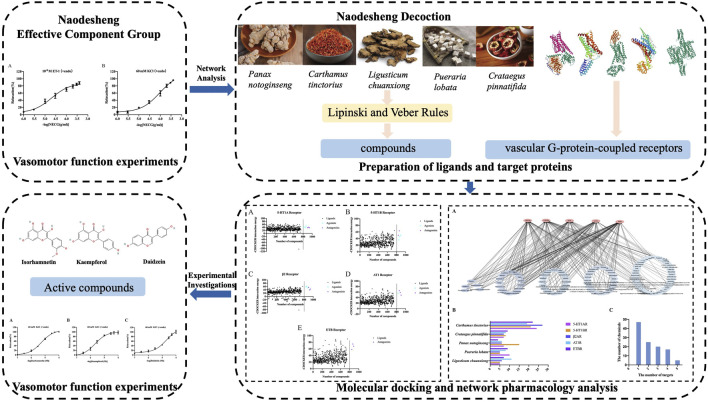
Flow chart.

In this study, we found that NECG could dilate the rat basilar artery precontracted using endothelin-1 (ET-1) (10^−8^ M) and KCl (60 mM) in a dose-dependent manner *in vitro*. However, the material basis of NDS on vascular function regulation remains largely undetermined. Therefore, the molecular docking approach was used for evaluating the synergistic regulation of the metabolites in NDS based on their interactions with vascular GPCRs, and a network of “NDS-GPCRs” was constructed for visualization. The material basis of NDS on vascular function regulation was subsequently analyzed and assessed using the isolated basilar artery ring relaxation and contraction model. This study may provide theoretical and experimental information for the development of new ischemic stroke-preventive drugs from NDS. The specific process analysis is shown in Figure 1.

## 2 Materials and methods

### 2.1 NECG preparation

The NECG was prepared by Professor Ruoyun Chen (Zhang et al., 2009) as before. In brief, NDS consists of five Chinese botanical drugs: *P. notoginseng* (Burk.) F. H. Chen (Araliaceae, *Notoginseng radix et rhizoma*)*,* 312 g; *L. chuanxiong* Hort. (Umbelliferae, *Chuanxiong rhizoma*), 312 g; *C. tinctorius* L., (Asteraceae, *C. flos*), 364 g; *P. lobata* (Willd.) Ohwi (Fabaceae, *Pueraria*e *radix*), 1,044 g; and *C. pinnatifida* Bge. (Rosaceae, *C. fructus*), 628 g; five medicinal materials were mixed in proportion, crushed with a blender, then extracted with 95% ethanol three times, 2 h each time, and all the extracts were mixed and concentrated into the total extract. The total extract was separated by macroporous resin, and the percentage of methanol was increased from 30% to 60% and then to 90% at room temperature. The 30% fraction (586.2 g) and 60% fraction (293.1 g) were collected and mixed into the NECG at 2:1 by weight. The freeze-dried samples were stored under 20°C for further tests. The quality control analysis of the NECG was performed using high-performance liquid chromatography (HPLC)-DAD (Supplementary Figures S1–4; Supplementary Tables S1, 2). Analytical HPLC was performed on an Agilent 1,200 instrument using a TSK gel ODS-100S column (250 × 4.6 mm, 5 μm) with a flow rate of 1.0 mL/min, and the gradient program of MeCN/0.1% HAc in H_2_O was 5:95 (t = 0 min), 10:90 (t = 25 min), 80:20 (t = 85 min), 90:10 (t = 86 min), and 90:10 (t = 96 min). HPLC-grade acetonitrile and other analytical-grade reagents were purchased from Beijing Chemical Reagent Co., Ltd. (Beijing, China). The 30% (1.0 mg/mL) and 60% (1.0 mg/mL) fractions and the marker compound (50 μg/mL, puerarin) purchased from Shifang Jubang Plant Raw Materials Co., Ltd (Deyang, China) were analyzed. The extraction and chemical analysis of the NECG complies with the standards established in the ConPhyMP statement (Heinrich et al., 2022).

### 2.2 Chemicals and reagents

Metabolites in NDS including isorhamnetin (YA0422YA14), genistein (Y25M6Y1), citric acid (B21313), and ursolic acid (B21403) were provided by Yuanye Biotechnology Co., Ltd. (Shanghai, China); kaempferol (20090615) was provided by Huike Plant Development Co., Ltd. (Shanxi, China); quercetin (117,395) was provided by Yaoyuan Biotechnology Co., Ltd. (Chengdu, China); daidzein (RSD-070426) was provided by Renshou Pharmaceutical Co., Ltd.; ferulic acid (20071006) was provided by Baicao Plant Co., Ltd. (Xuancheng, China); and puerarin (20070723) was provided by Shifang Jubang Plant Raw Materials Co., Ltd. (Deyang, China). Acetylcholine (ACh) and ET-1 were purchased from Sigma, United States. Other reagents are all domestically pure and meet the requirements of each experiment.

### 2.3 Preparation of rat cerebral arterial rings

Animal care and handling were performed following the guidelines of the Institutional Animal Care and Use Committee of the Chinese Academy of Medical Science and Peking Union Medical College. Male Sprague–Dawley rats, weighing 240–260 g, were purchased from Beijing HFK Biotechnology Co., Ltd. (Beijing, China. Certificate no: 11401300027357). The animals were housed in cages with sufficient rat food and water and maintained under a temperature-controlled room (24°C ± 1°C) with a 12 h light/dark cycle.

The preparation and vasomotor experiment of rat cerebral arterial rings were performed as described previously (Yuan et al., 2014). In brief, the rats were euthanized by cervical dislocation, following which the brain tissue was immediately removed and immersed in ice-cold physiological saline solution (PSS) with the following composition (mM): NaCl 130, KCl 4.7, KH_2_PO_4_ 1.18, NaHCO_3_ 14.9, glucose 5.5, CaCl_2_ 1.6, MgSO_4_ 1.17, and ethylene diamine tetraacetic acid 0.026. The basilar arteries were isolated, followed by the removal of tissue debris, cut into strips (2–3 mm), and mounted with 40-μm diameter tungsten wire in a well-oxygenated (95% O_2_-5% CO_2_) bath containing 5.0 mL PSS at 37°C and equilibrated for 60 min at the resting tension. The PSS solution was changed every 20 min during the equilibration period. After equilibration, the arterial rings were given two constrictions with high K^+^ (60 mM) PSS (K-PSS). The composition of the K-PSS solution was (mM): NaCl 74.7, KCl 60, KH_2_PO_4_ 1.18, NaHCO_3_ 14.9, glucose 5.5, CaCl_2_ 1.6, MgSO_4_ 1.17, and ethylene diamine tetraacetic acid 0.026. Subsequently, the endothelial integrity was verified using ACh (20 μM). The tension of the basilar artery was recorded isometrically in a Multi Myograph System (Danish Myograph Technology, Aarhus, Denmark). The endothelium-intact rings inducing more than 60% vasodilation in the 60 mM K-PSS were considered endothelium intact.

### 2.4 Measurement of the effects of the NECG and metabolites of NDS on an ET-1- or high-KCl-precontracted cerebral basilar artery

The rat endothelium-intact basilar arterial rings were given stimulations with ET-1 (10^−8^ M) or high KCl (60 mM). After the plateau was attained, the NECG was added (1, 3, 10, 30, 100, 200, 300, and 400 μg/mL) to obtain the concentration–response curves.

Based on the results of molecular docking, the rat endothelium-intact basilar arterial rings were pretreated with KCl (60 mM) to induce a steady contraction in advance. When the contraction reached a plateau, the 10 μM metabolites were added into the buffer, and the relaxation rate of the cerebral basilar artery precontracted using KCl was investigated and calculated.

For the metabolites with more than 50% relaxing effects, the arterial rings were precontracted using 60 mM KCl, the vascular tension was recorded, and concentration–response curves of these metabolites were constructed at various dosages (0.1, 0.3, 1, 3, 10, 30, 50, and 100 μM).

### 2.5 Retrieval of the metabolites of NDS

The chemical metabolites of the five botanical drugs in NDS were retrieved from the China Natural Product Database (http://pharmdata.ncmi.cn), the Traditional Chinese Medicine Database (https://tcmsp-e.com), and Traditional Chinese Medicine Database@Taiwan (http://tcm.cmu.edu.tw).

### 2.6 Preparation of ligands and target proteins

The crystal structures of 5-HT1AR, 5-HT1BR, β2AR, AT1R, and ETBR were all retrieved from the RCSB Protein Data Bank (http://www.pdb.org/), and the corresponding PDB codes were 7E2Y, 4IAQ, 4LDE, 4YAY, and 6K1Q, respectively (Berman et al., 2000).

By using the Prepare Ligands tool in Discovery Studio 4.1, all the different conformations of the metabolites were generated to form a library of small ligand molecules. The target proteins were pretreated using the Prepare Protein tool to remove existing ligands and water of crystallization. The position and size of the active center were determined using the Define and Edit Binding Site module, according to the relevant literature of the target-binding positive ligand. For 5-HT1AR and 5-HT1BR, serotonin and dihydroergotamine were defined as the active ligand-binding pockets, respectively. 8-[(1*R*)-2-{[1,1-dimethyl-2-(2-methylphenyl)ethyl]amino}-1-hydroxyethyl]-5-hydroxy-2H-1,4-benzoxazin-3(4H)-one, 5,7-diethyl-1-{[2'-(1H-tetrazol-5-yl) biphenyl-4-yl]methyl}-3,4-dihydro-1,6-naphthyridin-2(1H)-one, and (2∼{*S*})-2-[[(2∼{*R*})-2-[(3,5-dimethylphenyl) carbonyl-methyl-amino]-3-(4-phenylphenyl) propanoyl]amino]-3-(1∼{H}-indol-3-yl) propanoic acid were defined as the binding sites of β2-AR, AT1R, and ETBR, respectively.

### 2.7 Molecular docking of receptor–ligand

All the docking studies were performed using CDOCKER, which adopts the CHARMM force field to dock flexible ligands into protein-binding sites. During docking period, the receptor remains rigid, while the ligand is flexible. Then, a random ligand conformation is generated from the initial ligand structure through high-temperature molecular dynamics and performs random rotation, which is suitable for docking research of any number of small molecule metabolites at the same time. The -CDOCKER interaction energy scores were subsequently obtained. The scores indicate the binding affinity between the receptors and the ligand conformations. The higher the -CDOCKER interaction energy scores are, the stronger the binding affinity of small molecules to proteins is. The top 50 metabolites with the highest score of each target were then selected for the result statistics.

The CDOCKER scores of agonists and antagonists, retrieved from the IUPHAR database (https://www.guidetopharmacology.org), were used to assess the potential activity of herbal metabolites in NDS. The following agonists and antagonists were used for molecular docking: 5-HT1AR (agonists: U92016A, vilazodone, and vortioxetine; antagonists: (S)-UH 301, robalzotan, and WAY-100635); 5-HT1BR (agonists: CP94253, eletriptan, and L-694,247; antagonists: GR-55562 and SB236057); β2-AR (agonists: formoterol, salmeterol, and zinterol; antagonists: carvedilol, propranolol, and timolol); AT1R (agonist: L-162,313; antagonists: candesartan, irbesartan, and valsartan); and ETBR (antagonists: bosentan, IRL2500, and k8794).

### 2.8 Correlation network of metabolite–target construction using Cytoscape

Correlation networks related to metabolites–targets were plotted using Cytoscape version 3.8.0, and the interactions were subsequently visualized.

### 2.9 Statistical analysis

Data are presented as the mean ± SEM. Statistical analyses were performed using GraphPad Prism 8.0. Non-linear regression followed by dose–response stimulation was used to calculate pEC_50_.

## 3 Results

### 3.1 NECG induces the relaxation of rat basilar arteries precontracted by ET-1 or high KCl

**FIGURE 2:**
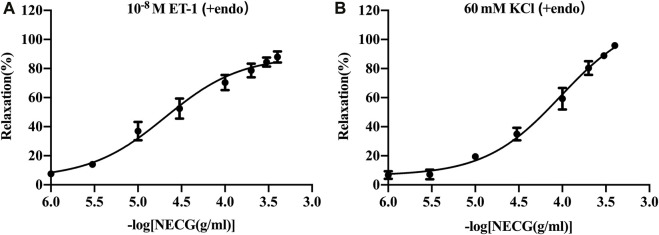
Vasorelaxant effects of the NECG on the rat endothelium-intact basilar artery preconstricted with ET-1 (10^−8^ M) or KCl (60 mM). **(A)** NECG dose-dependently relaxed ET-1 (10^−8^ M)-precontracted basilary artery rings. **(B)** NECG dose-dependently relaxed KCl (60 mM)-precontracted basilary artery rings. The results are presented as the mean ± SEM (*n* = 6).

The NECG caused a relaxation in ET-1 (10^−8^ M)- or high-KCl (60 mM)-precontracted rat basilar artery rings (Figure 2). The maximum relaxant effect (*E*_max_) of the NECG in the ET-1 (10^−8^ M) was 87.91% ± 3.82%. The pEC_50_ value was 4.69 ± 0.12 (Figure 2A). The NECG also relaxed basilar artery rings precontracted with KCl (60 mM) in a similar way (E_max_ = 95.89 ± 1.24%, pEC_50_ = 4.02 ± 0.10) (Figure 2B).

### 3.2 Construction and validation of the metabolite database of NDS

**FIGURE 3:**
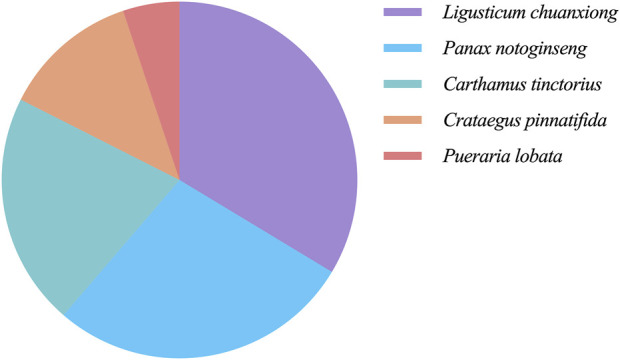
Proportion of the herbal metabolites in NDS.

In order to elucidate the material basis of the vascular regulatory function of NDS, 1,201 metabolites from the five botanical drugs of NDS were retrieved from three databases, and the number of metabolites in each medicinal material was determined, as shown in Figure 3. The metabolites were subsequently classified through the Cluster ligands function of Discovery Studio 4.1, and 10 types of metabolites in NDS were obtained. The distribution of these metabolites in different categories is provided in Table 1. The results showed that the top three metabolite number ranking categories in NDS were notoginsenoside R4 (23%), astragalin (17%), and limonene (16%). Nevertheless, it should be pointed out that the statistical results reflect the difference in the number of different types of metabolites instead of representing the relative content in the prescription.

**TABLE 1:** Classification of the cluster center and number of chemical metabolites in NDS.

Group	Cluster center	Number	Group	Cluster center	Number
A	Pentadecane	104	F	Furol	31
B	Dichloroaniline	6	G	Limonene	190
C	Nonacosanol	83	H	Notoginsenoside R4	272
D	Astragalin	201	I	Acetophenone	114
E	Butyric acid	129	J	4,7-Dihydroxy-3-butylphthalide	71
				In total	1,201

In order to determine the potentially active metabolites of NDS, we combined and counted the repeated metabolites in botanical drugs. A total of 51 metabolites were obtained from different kinds of botanical drugs (Supplementary Table S3). Among the metabolites with a variety of plant sources, most of them belong to the category of butyric acid, pentadecane, 4,7-dihydroxy-3-butylphthalide, and notoginsenoside R4. This result was similar to that obtained from the cluster analysis.

### 3.3 Molecular docking

Metabolites complying with Lipinski rules can obtain higher bioavailability during metabolism in organisms. Therefore, the 1,201 chemical metabolites retrieved by the above screening were analyzed by Lipinski and Veber rules, and finally, 777 metabolites were obtained.

**FIGURE 4:**
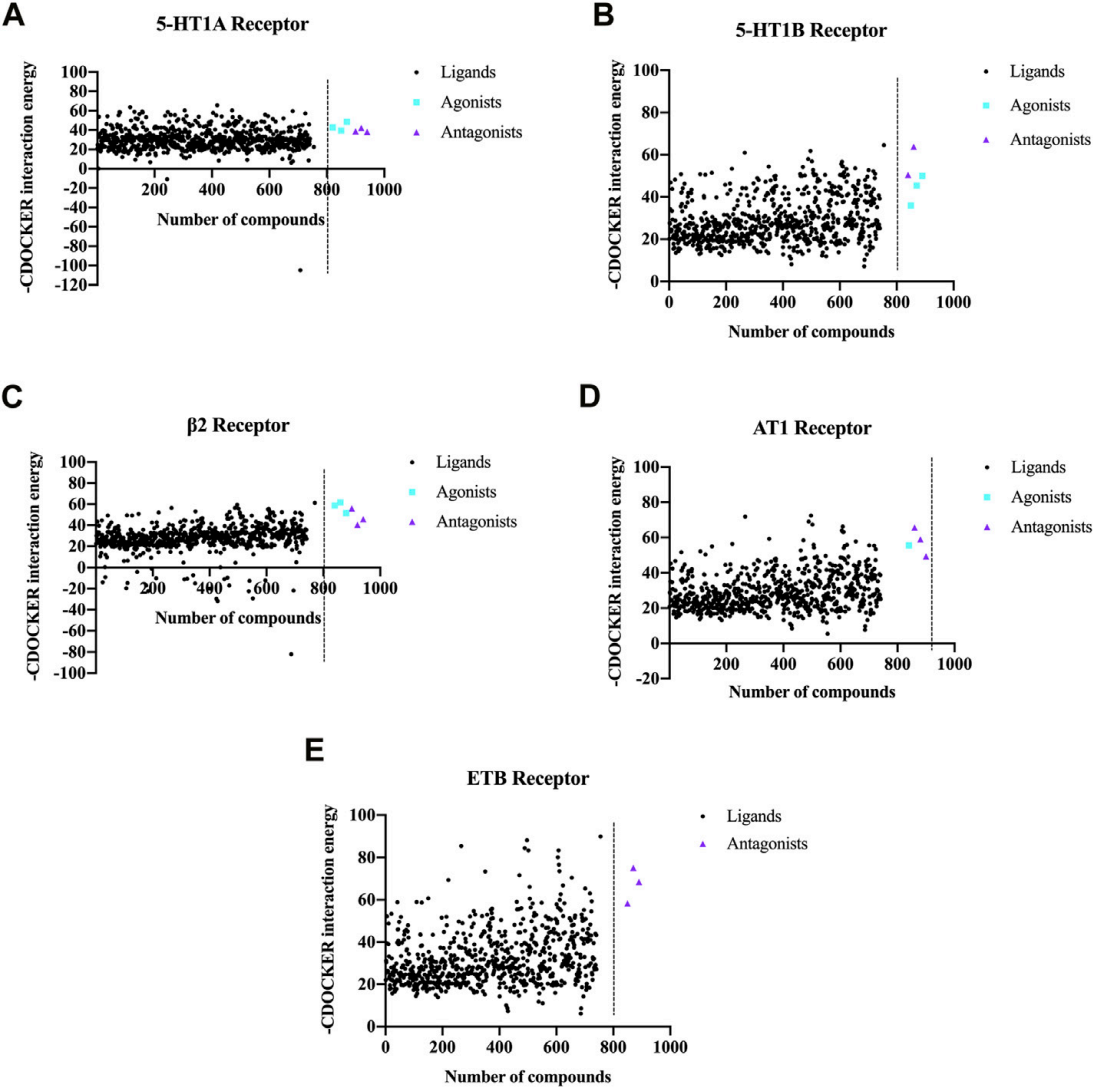
CDOCKER scores obtained by molecular docking of the chemical metabolites in NDS with the five vascular GPCR targets. The five vascular GPCR targets are **(A)** 5-HT1AR, **(B)** 5-HT1BR, **(C)** β2-AR, **(D)** AT1R, and **(E)** ETBR.

For each GPCR vasomotor target, the metabolites with the highest scores were selected to generate a scatter plot, as depicted in Figure 4. The CDOCKER scores of the agonists and antagonists of the GPCR targets are also shown in Figure 4. For each GPCR, partial metabolites exhibited higher scores than the agonists and antagonists, indicating that they have stronger binding affinity to the GPCR targets.

In order to analyze the overall situation of the ligand–receptor docking, the average value of each receptor-positive drug was calculated, and the total average value of five receptor-positive drugs is 51.68 (Supplementary Table S4). Among the top 50 metabolites, the proportions of metabolites whose CDOCKER scores are higher than the average scores of the positive drugs are shown in Table 2, and more than 20% of NDS metabolites had higher CDOCKER scores than the average scores of the GPCR agonists and antagonists. Therefore, these results indicated that NDS can comprehensively regulate vascular tension through the interaction between various chemical metabolites of NDS and different vascular GPCR targets in the composite target network.

**TABLE 2:** Proportion of the metabolites in top 50 chemical metabolites of NDS excelled the average score of positive drugs interacting with the five vascular GPCRs.

(%)	5-HT1AR	5-HT1BR	β2AR	AT1R	ETBR
>Positive drug mean ratio	100.00	80.00	24.00	22.00	26.00

### 3.4 Construction and analysis of a metabolite–target interaction network of NDS

**FIGURE 5:**
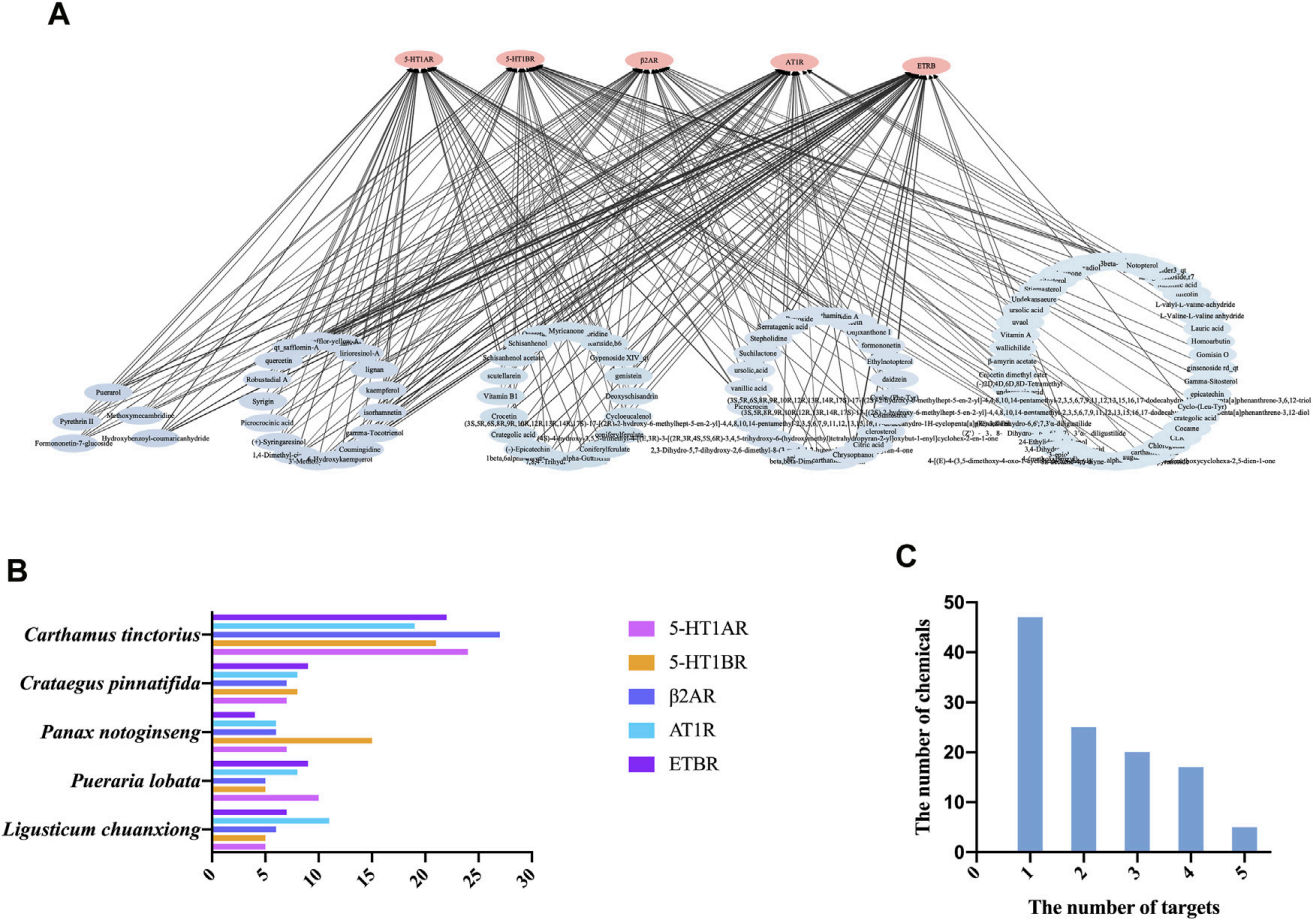
**(A)** Chemical composition (score in the top 50) target interaction network of NDS. **(B)** Enrichment of the 50 top-scoring metabolites with high docking scores interacting with each of the vascular GPCR targets, which were sorted and calculated according to their herb origin in NDS. **(C)** Number of top 50 scoring metabolites interacting with multiple vascular GPCR targets.

The 50 top-scoring metabolites targeting each vascular GPCR were then collected and deduplicated to obtain 114 metabolites for visual analysis using Cytoscape version 3.8.0. As shown in Figure 5A, the thickness of the black interaction line represents the binding affinity of the metabolites and the targets. The thicker the line, the greater the binding ability, while the composition of the intermediate region indicates that it is connected to a plurality of targets. The results revealed that the metabolite–target network comprised 250 edges, 119 nodes, 114 metabolites (after the removal of duplicates), and 5 targets. It is speculated that these metabolites play a role in vasodilation as the main active metabolites of NDS.

In order to investigate the relationship between the chemical metabolites of the five botanical drugs of NDS and five vascular GPCR targets, the 50 top-scoring metabolites with the highest docking scores of each GPCR target were classified and sorted according to their herbal sources (Figure 5B). The top three medicinal materials were *C. tinctorius*, *C. pinnatifida*, and *P. notoginseng*.

Subsequently, the distribution framework was further statistically analyzed. Several of the 50 top-scoring metabolites were found to interact with multiple targets, including 5-HT1AR, 5-HT1BR, AT1R, β2-AR, and ETBR. As depicted in Figure 5C, five metabolites of NDS, namely, formononetin-7-glucoside, hydroxybenzoyl-coumaric anhydride, methoxymecambridine, puerarol, and pyrethrin II, interacted with all the five vascular GPCRs, while 17 metabolites, including isorhamnetin, kaempferol, and quercetin, interacted with four targets. On the other hand, the existing 20 metabolites, including vitamin B1 and genistein, interacted with three GPCR targets, and 25 metabolites, including citric acid, daidzein, and ursolic acid, interacted with two of the GPCR targets.

### 3.5 Further investigation of NDS metabolites in the KCl-precontracted rat cerebral basilar artery according to molecular docking results

To further investigate the above molecular docking results *in vitro*, a preliminary screening experiment was carried out in the KCl-precontracted rat endothelium-intact cerebral basilar artery. According to the total average CDOCKER scores of 51.68 for five receptor-positive drugs (Supplementary Table S4), metabolites with average docking scores higher than 51.68, such as isorhamnetin, kaempferol, and genistein, were determined as metabolites with high docking scores. Metabolites with average docking scores lower than 41.59 (Supplementary Table S4, the lowest average CDOCKER score in five receptor-positive drugs), such as citric acid, ferulic acid, and ursolic acid, were determined as metabolites with low docking scores. Furthermore, metabolites with average docking scores among the two values, such as quercetin and daidzein, were determined as metabolites with medium docking scores. Therefore, metabolites with high, medium, and low docking scores and having more activity reports in NDS were chosen for the vasomotor function experiments (Table 3). Relaxation activities of each metabolite at 10 μM on the endothelium-intact basilar artery precontracted using 60 mM KCl are shown in Table 4. Metabolites with negative relaxation values were citric acid and ferulic acid. Moreover, the other six metabolites displayed a certain relaxation effect in the rat cerebral basilar artery precontracted using KCl, of which the monomers with a relaxation rate of more than 50% were isorhamnetin, kaempferol, quercetin, and daidzein. It has been reported that quercetin has a significant vascular relaxation effect on 60 mM KCl-precontracted intact basilar artery rings *in vitro*; its maximum diastolic rate is 98.82% ± 0.53% (Yuan et al., 2018). We further investigated the dose–effect characteristic of the other three metabolites.

**TABLE 3:** Overview of the CDOCKER scores of eight metabolites in NDS.

Group	No.	Metabolite	5-HT1AR	5-HT1BR	β2R	AT1R	ETBR
High score	1	Isorhamnetin	63.62	43.13	52.09	72.45	89.94
	2	Kaempferol	59.27	41.14	49.50	67.39	84.49
	3	Genistein	57.21	40.74	42.79	58.43	65.42
Medium score	4	Quercetin	49.49	41.07	49.07	54.10	58.65
	5	Daidzein	54.03	39.35	40.97	43.92	62.60
Low score	6	Citric acid	29.63	21.72	31.93	46.43	69.36
	7	Ferulic acid	34.16	26.93	32.22	35.22	43.95
	8	Ursolic acid	28.32	49.81	−12.54	44.46	51.35

**TABLE 4:** Metabolite of NDS according to the relaxant rate of the metabolites on the endothelium-intact rat basilar artery precontracted with KCl (60 mM).

Metabolite	Vasodilation rate (%)
Isorhamnetin	52.89
Kaempferol	95.34
Genistein	36.00
Quercetin	71.08
Daidzein	53.18
Citric acid	−12.75
Ferulic acid	−9.82
Ursolic acid	9.31

**FIGURE 6:**
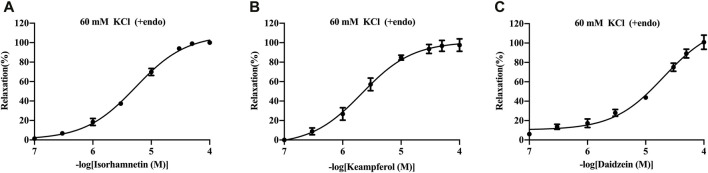
Vasorelaxant effects of isorhamnetin **(A)**, kaempferol **(B)**, and daidzein **(C)** on the endothelium-intact rat basilar artery precontracted with KCl (60 mM). The results are presented as the mean ± SEM (*n* = 3).

The basilar arteries were precontracted with 60 mM KCl and then incubated with the three metabolites of isorhamnetin, kaempferol, and daidzein at various dosages (0.1, 0.3, 1, 3, 10, 30, 50, and 100 μM), and the vascular tension was recorded. Among the three metabolites, daidzein exhibited a stronger vasorelaxant effect on the rat cerebral basilar artery precontracted with KCl than the other two metabolites, which is 100.85% ± 7.34% (Figure 6; Table 5).

**TABLE 5:** Vasorelaxant effects of metabolites on the rat endothelium-intact basilar artery precontracted with KCl (60 mM).

Metabolite	pEC_50_ (μM)	Maximum vasodilation rate (%)
Isorhamnetin	5.27 ± 0.04	100.10 ± 0.45
Kaempferol	5.67 ± 0.08	97.57 ± 6.43
Daidzein	4.69 ± 0.10	100.85 ± 7.34

## 4 Discussion

In the past 2 decades, generally, it can be assumed that a single-target drug has a modest therapeutic efficacy on multi-factorial complex diseases. Therefore, multi-target agents have attracted attention. With the all-round development of systems biology and network analysis, several studies have applied the network analysis approach to analyze the TCM with multi-component and multi-target (Zhang et al., 2019). It has been suggested that NDS can improve cerebrovascular circulation, relax and widen the cerebral vessels, improve cognition, and promote neurological recovery (Cai et al., 2008; Chen et al., 2008; Guo and Yang, 2009; Zhang et al., 2021). Therefore, there is no doubt that NDS is effective in the treatment of ischemic stroke. However, which metabolites in NDS may play a role in the treatment of ischemic stroke has not been studied. Considering the interconnectedness between metabolites and vasomotor receptors in ischemic stroke, we aim to explore the therapeutic effect of NDS on ischemic stroke through network pharmacological analysis and experimental investigation.

In the current study, the vasodilator effect of the NECG was demonstrated in the rat cerebral basilar arteries precontracted using ET-1 or high KCl. When suffering from different types of cerebral ischemia, the regulation of vascular tone can be achieved by activating specific intracellular signal transduction pathways in cerebral artery smooth muscle cells, thereby upregulating the expression of specific GPCRs in vascular smooth muscle cells (VSMCs) (Edvinsson and Povlsen, 2011). Thus, we conducted molecular docking to determine whether the metabolites of NDS could make up the synergistic effect and regulate the activity of vascular GPCRs in response to vessel tone. A database containing 777 NDS metabolites with different chemical structures was constructed and analyzed. The interaction between the chemical metabolites of NDS and five vascular GPCRs, namely, 5-HT1AR, 5-HT1BR, β2-AR, AT1R, and ETBR, was investigated by molecular docking. The top 50 metabolites with higher docking scores for each GPCR target were selected, and the plant source analyses were performed to determine the five medicinal materials of NDS to regulate vascular tension by modulating GPCR activity. Therefore, our experiments showed that GPCR may be one of the targets for the NECG modulate to regulate vascular tension.

The non-hierarchical clustering of spectral clustering was used for intuitive description. Cluster analysis is a multi-category analysis process composed of similar objects. According to statistics, it is found that most of the metabolites belonging to the same category showed similar pharmacological activities; for example, puerarin, a metabolite from group D (astragalin) (Table 1), exhibited a cerebral vasodilation effect on U46619 (100 nM)-preconstricted rat basilar artery rings, and its IC50 is 304 ± 49 μM; both daidzein and daidzin also evoked concentration-dependent relaxation (Deng et al., 2012). Furthermore, hyperin (1–100 μM) also showed vasodilatory activity in a concentration-dependent manner after the basilar artery segment was precontracted with 0.1 μM U46619 (Fan et al., 2011). Our research yielded a total of 51 metabolites that appeared in different kinds of plant materials repeatedly (Supplementary Table S3). After finishing the categorization of these 51 metabolites, butyric acid, pentadecane, 4,7-dihydroxy-3-butylphthalide, and notoginsenoside R4 were found to rank in the top field, suggesting that metabolites belonging to these chemical categories in NDS may play a critical role in exerting their pharmacological activities.

Based on the molecular docking results, the plant material sources of the top 50 high-scoring metabolites corresponding to the five targets were further classified and calculated. The top three plant materials were *C. tinctorius*, *C. pinnatifida*, and *P. notoginseng*. These botanical drugs and their metabolites have been widely found to have vascular tone regulating function. It has been reported that hydroxysafflor yellow A (HSYA), the representative metabolite of *C. tinctorius*, has vasodilation activity in both rat cerebral arteries (Sun et al., 2018) and mesenteric arteries (Yang et al., 2020). Hawthorn water extract also attenuated the vasoconstriction of the rat mesenteric artery induced using U46619 (30 nM), phenylephrine (1 μM), and KCl (60 mM) and improved endothelial-dependent relaxation (Chen et al., 1998). The vasodilation effect of the ethanol extract of *P. notoginseng* (PN95) is related to the modulation of the NO/sGC/cGMP and β2-adrenergic receptor pathway (Loh et al., 2019). Notoginseng triol saponins have been known to facilitate the expression of VEGF, Ang1, and their receptors, thereby resulting in increased cerebral blood supply (Hui et al., 2017). Therefore, *C. tinctorius*, *C. pinnatifida*, and *P. notoginseng* may play a critical role in the vascular tone regulatory effects of NDS. However, the subtle metabolite–target interaction network regulation of vascular tone in NDS still remains to be further investigated.

By searching for literature, we found that most of the metabolites of NDS that target multiple vascular GPCRs were reported to have the ischemic stroke damage alleviating activity. For instance, vitamin B1 binding to three GPCR targets was reported to reduce thiamine, one of the predictors of early cognitive impairment in patients with acute cerebral infarction (Feng et al., 2020). The (+)-syringaresinol in *C. tinctorius* binding to four GPCRs could induce vasorelaxation by increasing NO production in endothelial cells (Chung et al., 2012). Interestingly, five metabolites in NDS, namely, formononetin-7-glucoside, hydroxybenzoyl-coumaric anhydride, methoxymecambridine, puerarol, and pyrethrin II, all bind to five vascular GPCRs and have high docking scores, but few studies involving their effects in vasodilation and ischemic stroke could be found. Hence, the effects of these five metabolites on ischemic stroke deserve to be further investigated. Our findings suggest that metabolites targeting vascular GPCRs may mediate the regulatory effects of NDS on vascular tone and provide a certain material basis of NDS for the ischemic stroke treatment. However, further *in vivo* experiment is needed to confirm these results.

The basilar cerebral artery is one of the arteries that provide oxygen-rich blood to the brain, and it has a significant impact on the resistance of cerebral blood vessels (Deng et al., 2012). Therefore, to further validate the material basis of the NDS in vessel vasodilatation, the vasodilation effect of the virtual screened active metabolites was tested in the isolated cerebral basilar artery rings precontracted with KCl. The results illustrated that among the 50 top-scoring metabolites, isorhamnetin, kaempferol, and daidzein, which exhibited high affinity with multiple GPCRs, showed good *in vitro* vasodilation activity on the basilar artery precontracted using KCl, suggesting that the docking results agreed with the vasodilation activity. Coinciding with our results, it has been reported that isorhamnetin from *C. pinnatifida*, which bound with four vascular GPCR targets, exerts a vasorelaxant effect via regulating the endothelial NO/_s_GC/cGMP pathway and cyclooxygenase pathway (Zhao et al., 2010). Clinical studies have shown that a higher intake of isorhamnetin-rich products could possibly result in decreased blood pressure (BP) in coronary artery disease (CAD) patients (Popiolek-Kalisz et al., 2022). Kaempferol as the active metabolite of *C. tinctorius,* which bound with four vascular GPCR targets too, has been shown to possess the relaxing effect of the isolated rat aortic ring and pulmonary artery (Xu et al., 2008; Mahobiya et al., 2018). Furthermore, daidzein from *P. lobata* has been reported to vasodilate the rat basilar artery by inhibiting Ca^2+^ influx and activating the BKCa channel in smooth muscle cells (Torregrosa et al., 2003; Zhang et al., 2010). Furthermore, quercetin, a common chemical metabolite of *C. tinctorius*, *C. pinnatifida*, and *P. notoginseng*, had been found to have vasodilatory effects on the cerebral basilar artery precontracted with KCl, ET-1, and 5-HT (Yuan et al., 2018).

From a chemical point of view, isorhamnetin, kaempferol, daidzein, and quercetin all belong to the group of flavonoids. Randomized controlled trials and other population-based studies suggest that many flavonoids contribute to inhibiting oxidation, alleviating inflammation, relieving pain, enhancing heart metabolism, and exerting vascular protective effects (Knekt et al., 2002; Bobe et al., 2010; Javadi et al., 2017; Ferraz et al., 2020). Epidemiological studies have found that the intake of flavonoids is associated with a decreased risk of cardiovascular diseases (CVDs) (Wang et al., 2014). Arts and Hollman (2005) evaluated the results of 12 cohort studies on flavonoid intake and coronary artery disease, as well as five cohort studies on flavonoid intake and stroke risk, with evidence of protective effects (Arts and Hollman, 2005). Additionally, the vascular relaxation and vasodilative nitric oxide formation promoted by flavonoids could partly explain the ability of many of these substances to reduce arterial blood pressure (Taubert et al., 2007). Relieving arterial occlusion and restoring cerebral blood flow are the ultimate therapeutic management for ischemic stroke and cerebral infarction, respectively (Prabhakaran et al., 2015). Due to the less number of drugs currently available for the clinical treatment of ischemic stroke, it is of great significance to find the potential material basis for the treatment of ischemic stroke. These clinical data further support our findings concerning the vasodilation effects of isorhamnetin, kaempferol, and daidzein.

This study has certain limitations. First, the public database may not include some compounds and target genes due to the universality of network pharmacology research. Second, we found that isorhamnetin, kaempferol, and daidzein dilated blood vessels *in vitro*; further studies with an expanded sample size and *in vivo* experiments are needed, including clinical studies, to determine the properties and full active potential of the active metabolites.

In conclusion, this study demonstrated that NECG was capable to relax the rat cerebral artery. NDS consists of various phytochemicals and originates from multiple plant sources. Thus, NDS might regulate cerebrovascular function through the interactions between the metabolites of NDS and the vascular GPCRs. In this study, the potential main metabolites with medium and high binding energy scores show good vasodilation effects through the molecular docking and *in vitro* experiment results, suggesting that molecular docking and network analysis are suitable for exploring the material basis of NDS in the prevention and treatment of stroke.

## Data Availability

The datasets presented in this study can be found in online repositories. The names of the repository/repositories and accession number(s) can be found in the article/Supplementary Material.
