# Supplementary material for: Naodesheng decoction regulating vascular function via G-protein-coupled receptors: network analysis and experimental investigations
Source: Front Pharmacol. 2024 Mar 12;15:1355169. doi: 10.3389/fphar.2024.1355169 (PMC10963398; doi:10.3389/fphar.2024.1355169)
Supplement: Supplementary file 1 [file DataSheet1.pdf]

Supplementary Material

Naodesheng Decoction Regulating Vascular Function via G-protein Coupled Receptors: Network Analysis and Experimental Investigations

Shuhan Chen[1], Ziran Niu[1,2], Yanjia Shen[1], Wendan Lu[1], Jiaying Zhao[1], Huilin Yang[1], Minmin Guo[1], Li Zhang[1], Ruifang Zheng[3] ,Guanhua Du[1*], Li Li[1*]

*** Correspondence:** Corresponding Author:

Dr. Guanhua Du
dugh@imm.ac.cn

Dr. Li Li
lili@imm.ac.cn

1 Supplementary Figures and Tables

1.1 Supplementary Figures

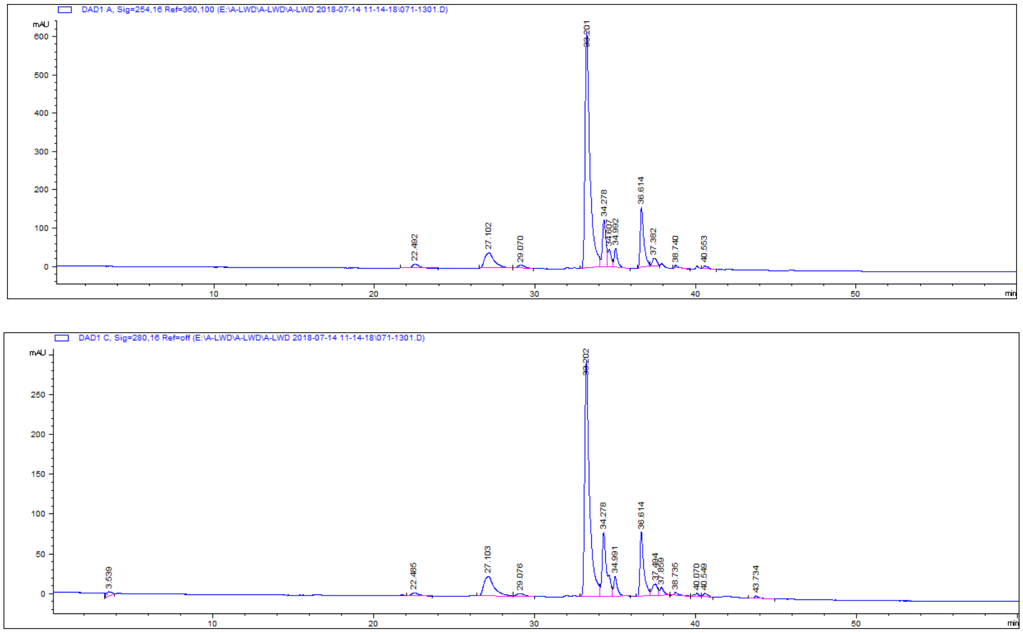

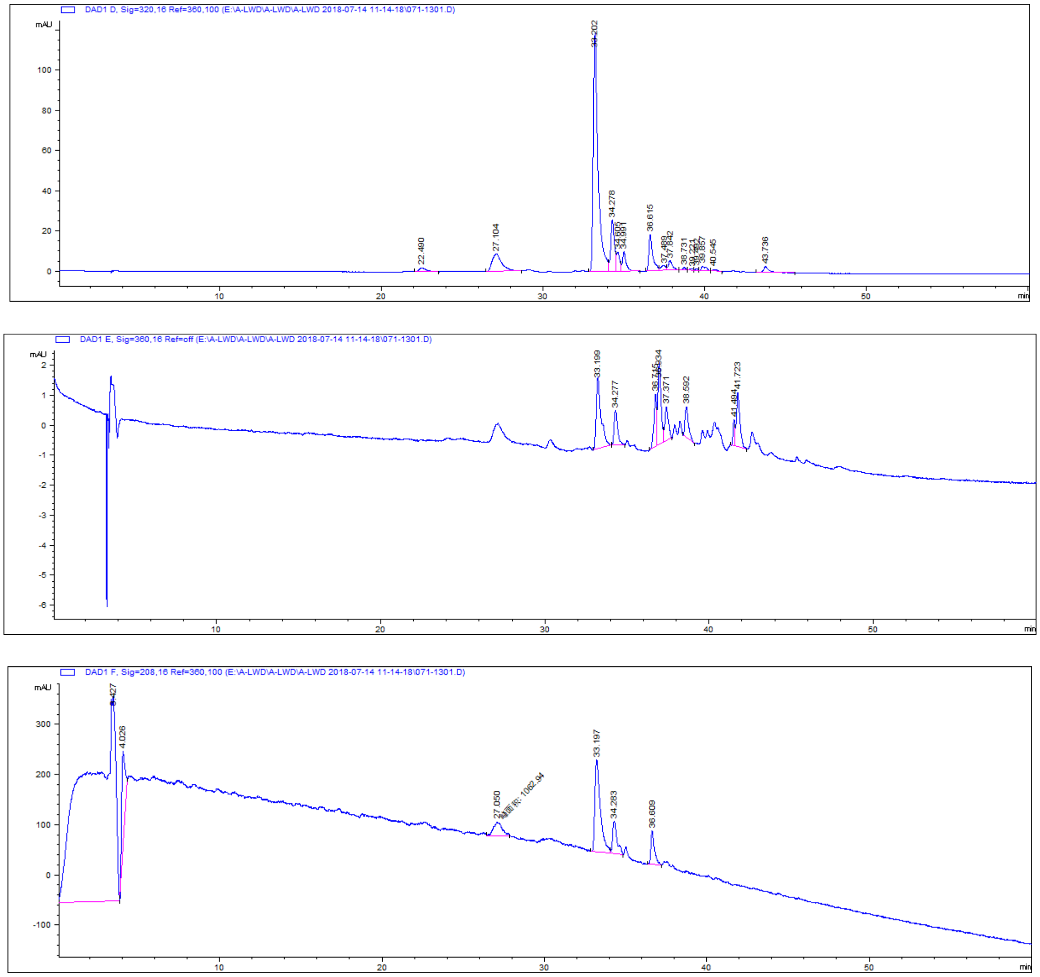

Supplementary Figure 1. HPLC/DAD chromatograms of the 30% fraction monitored at 254, 280, 320, 360, and 208 nm.

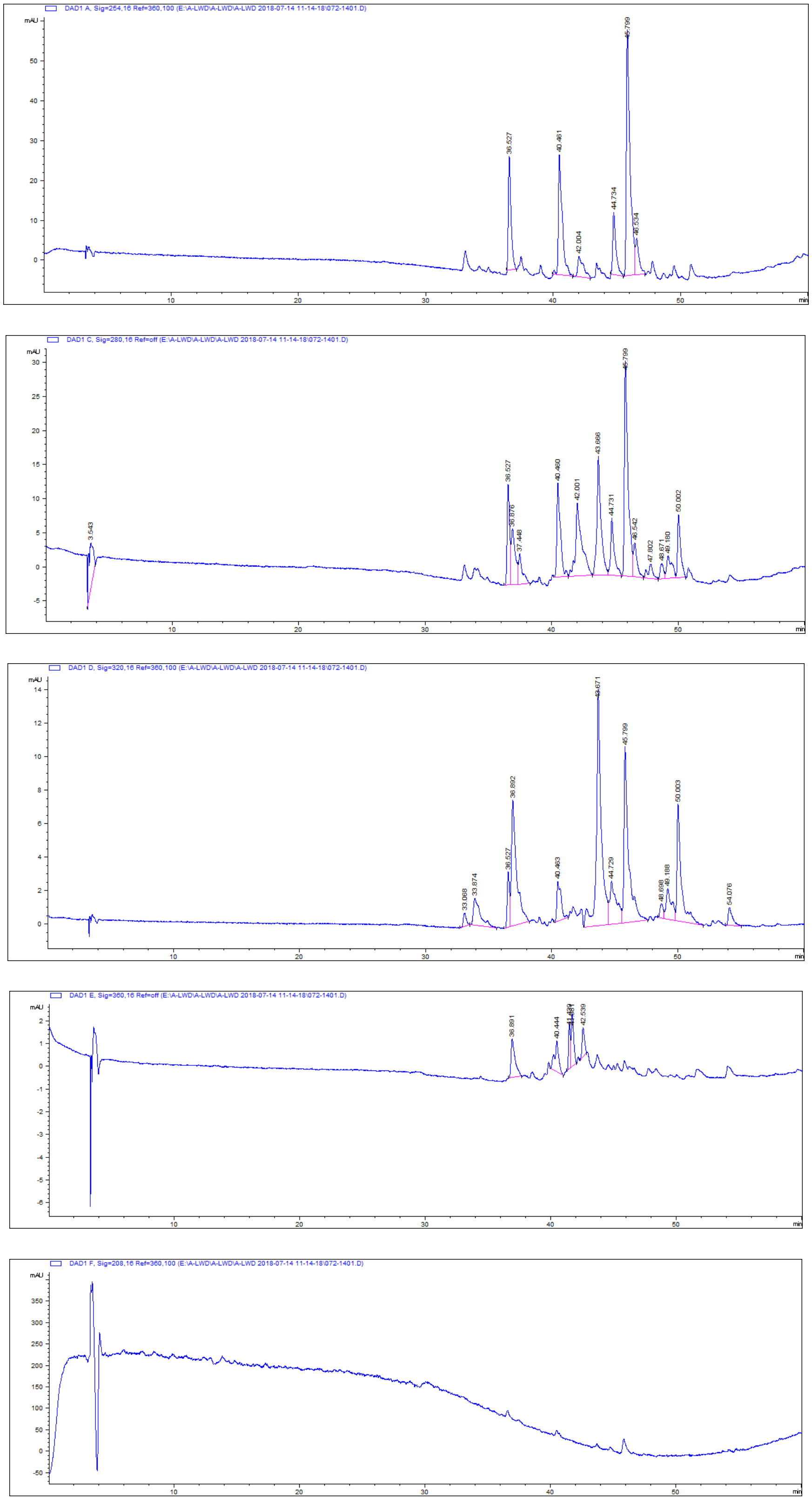

Supplementary Figure 2. HPLC/DAD chromatograms of the 60% fraction monitored at 254, 280, 320, 360, and 208 nm.

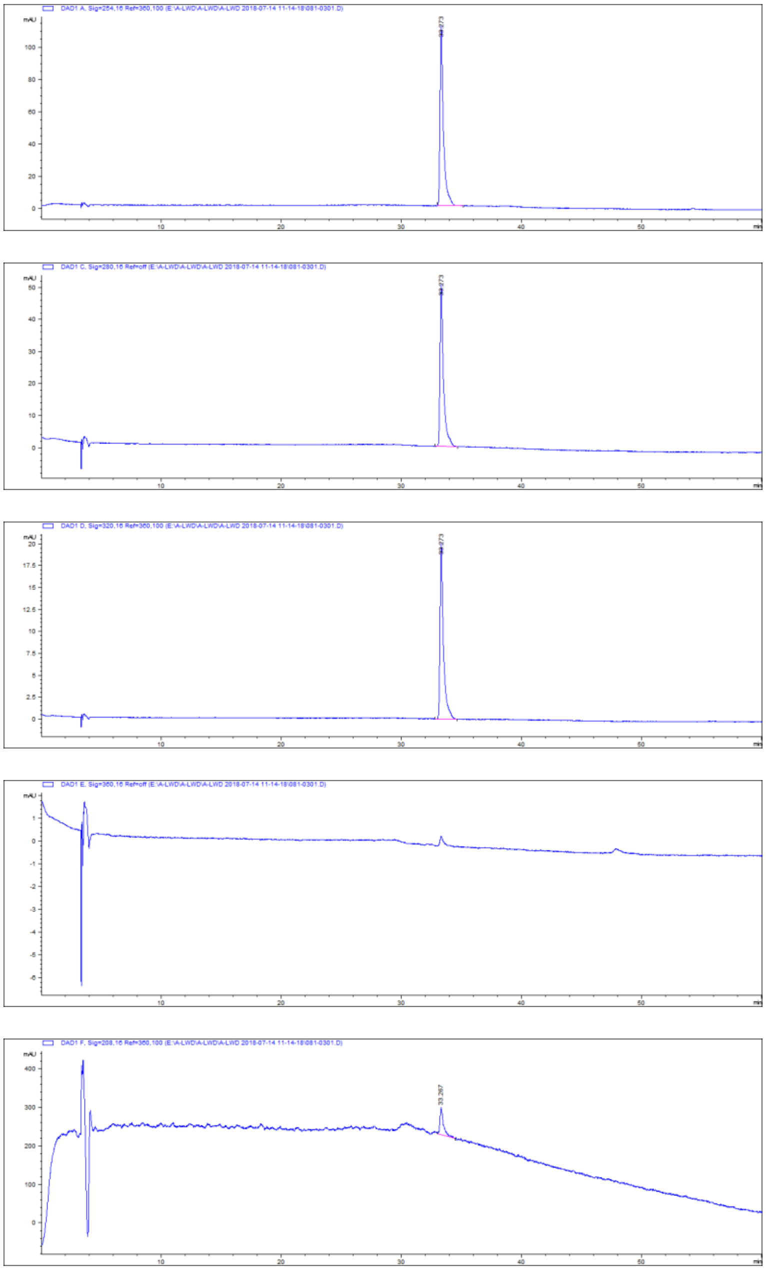

Supplementary Figure 3. HPLC/DAD chromatograms of the marker compound puerarin monitored at 254, 280, 320, 360, and 208 nm.

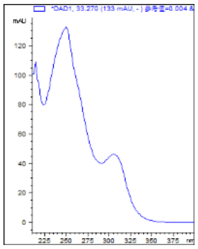

Supplementary Figure 4. The UV spectra of the marker compound puerarin detected by on line HPLC-DAD.

1.2 Supplementary Tables

Supplementary Table 1. The UV spectra of the major peaks of the 30% fraction

Peak t_R (min)	33.201	34.278	36.614
UV spectra[a]	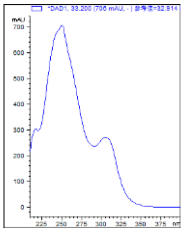 Marker compound puerarin	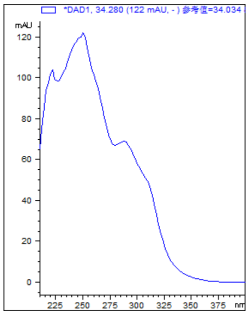	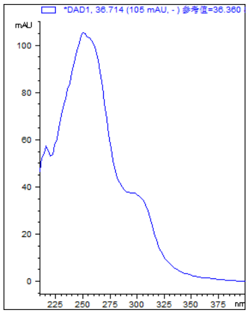

[a] detected by on line HPLC-DAD

Supplementary Table 2. The UV spectra of the major peaks of the 60% fraction

Peak t_R (min)	36.527	40.461	44.734
UV spectra[a]	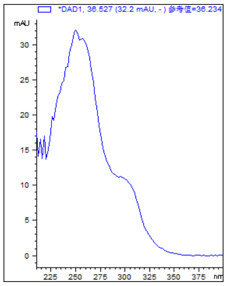	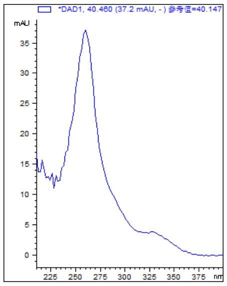	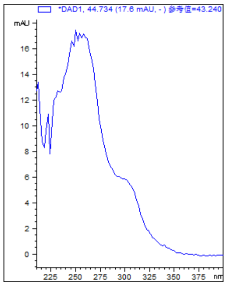
Peaks t_R (min)	45.799	50.002	
UV spectra[a]	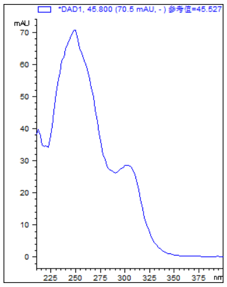	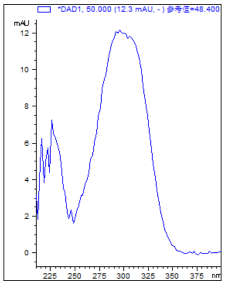	

[a] detected by on line HPLC-DAD

Supplementary Table 3. The Overlapping chemicals derived from different botanical drugs in NDS decoction

Metabolite	Plant source	Chemical classification	Metabolite	Plant source	Chemical classification
1-hexadecene	*Ligusticum chuanxiong, Carthamus tinctorius*	A	stearic acid	*Ligusticum chuanxiong, Panax notoginseng, Carthamus tinctorius, Crataegus pinnatifida*	E
Ethylpalmitate	*Ligusticum chuanxiong, Panax notoginseng*	A	succinic acid	*Carthamus tinctorius, Crataegus pinnatifida*	E
hexanal	*Ligusticum chuanxiong, Panax notoginseng*	A	zoomaric acid	*Carthamus tinctorius, Panax notoginseng*	E
Methyl hexadecanate	*Ligusticum chuanxiong, Panax notoginseng*	A	Methyllinolenate	*Ligusticum chuanxiong, Carthamus tinctorius*	F
Pentadecane	*Ligusticum chuanxiong, Panax notoginseng*	A	(+)-Terpinen-4-ol	*Ligusticum chuanxiong, Carthamus tinctorius*	G
Nonanal	*Ligusticum chuanxiong, Carthamus tinctorius*	A	β-elemene	*Ligusticum chuanxiong, Panax notoginseng*	G
Stearin	*Carthamus tinctorius, Crataegus pinnatifida*	A	Germacrene D	*Ligusticum chuanxiong, Panax notoginseng*	G
tetradecane	*Ligusticum chuanxiong, Panax notoginseng*	A	spathulenol	*Ligusticum chuanxiong, Panax notoginseng*	G
(-)-spathulenol	*Ligusticum chuanxiong, Panax*	C	20-Hexadecanoylingenol	*Ligusticum chuanxiong, Panax*	H

	notoginseng			*notoginseng, Carthamus tinctorius, Crataegus pinnatifida*	
Dauricine	*Pueraria lobata, Panax notoginseng*	D	Gamma-Sitosterol	*Ligusticum chuanxiong, Panax notoginseng, Carthamus tinctorius, Pueraria lobata, Crataegus pinnatifida*	H
Kaempferol	*Carthamus tinctorius, Crataegus pinnatifida*	D	oleanolic acid	*Panax notoginseng, Crataegus pinnatifida*	H
Pancratistatin	*Panax notoginseng, Carthamus tinctorius*	D	Senlciphylline	*Ligusticum chuanxiong, Notoginseng Radix et*	H
quercetin	*Panax notoginseng, Carthamus tinctorius, Crataegus pinnatifida*	D	Sitogluside	*Ligusticum chuanxiong, Panax notoginseng, Carthamus tinctorius, Pueraria lobata*	H
rutin	*Carthamus tinctorius, Crataegus pinnatifida*	D	sitosterol	*Ligusticum chuanxiong, Crataegus pinnatifida*	H
Vitamin- G	*Carthamus tinctorius, Crataegus pinnatifida*	D	Stigmasterol	*Panax notoginseng, Carthamus tinctorius, Crataegus pinnatifida*	H
Adenosine	*Ligusticum chuanxiong, Carthamus tinctorius*	D	Caffeic acid dimethyl ether	*Ligusticum chuanxiong, Crataegus pinnatifida*	I
14-Methyl hexadecanoic acid	*Ligusticum chuanxiong, Panax notoginseng*	E	Ethyl-p-digallate	*Ligusticum chuanxiong, Panax notoginseng*	I

Arachidic acid	*Pueraria lobata, Carthamus tinctorius*	E	3-caffeoylquinic,acid	*Ligusticum chuanxiong, Crataegus pinnatifida*	J
Daturic acid	*Carthamus tinctorius, Panax notoginseng*	E	3-o-caffeoylquinic,acid	*Ligusticum chuanxiong, Crataegus pinnatifida*	J
Hexadecanoic acid	*Ligusticum chuanxiong, Panax notoginseng, Carthamus tinctorius, Crataegus pinnatifida*	E	adenine	*Ligusticum chuanxiong, Carthamus tinctorius*	J
hexanoic acid	*Carthamus tinctorius, Panax notoginseng*	E	caffeic acid	*Carthamus tinctorius, Carthamus tinctorius, Crataegus pinnatifida*	J
Hexenal	*Ligusticum chuanxiong, Panax notoginseng*	E	chlorogenetic,acid	*Ligusticum chuanxiong, Crataegus pinnatifida*	J
lauric acid	*Carthamus tinctorius, Panax notoginseng*	E	Chlorogenic acid	*Ligusticum chuanxiong, Crataegus pinnatifida*	J
Linolenic acid	*Carthamus tinctorius, Crataegus pinnatifida*	E	chlorogenic,acid	*Ligusticum chuanxiong, Crataegus pinnatifida*	J
Linoleyl acetate	*Carthamus tinctorius, Crataegus pinnatifida*	E	neo-chlorogenic,acid	*Ligusticum chuanxiong, Crataegus pinnatifida*	J
Myristic acid	*Carthamus tinctorius, Panax notoginseng*	E			

A: Pentadecane; B: Dichloroaniline; C: Nonacosanol; D: Astragalin; E: Butyric, acid; F: Furol; G: Limonene; H: Notoginsenoside R4; I: Acetophenone; J: 4,7-Dihydroxy-3-butylphthalide.

Supplementary Table 4. The CDOCKER scores of five vascular GPCRs agonists and antagonists

Receptor	Type	Agonist/Antagonist	Score	Average docking score
5-HT1AR	Agonists	U92016A	42.67	41.59
		Vilazodone	48.64	
		Vortioxetine	39.33	
	Antagonists	(S)-UH 301	38.15	
		Robalzotan	38.65	
		WAY-100635	42.08	
5-HT1BR	Agonists	CP94253	36.00	49.12
		Eletriptan	45.41	
		L-694,247	49.92	
	Antagonists	GR-55562	50.50	
		SB236057	63.78	
β2-AR	Agonists	Formoterol	58.74	52.32
		Salmeterol	61.53	
		Zinterol	51.63	
	Antagonists	Carvedilol	55.99	
		Propranolol	40.33	
		Timolol	45.68	
AT1R	Agonists	L-162,313	55.56	57.38
	Antagonists	Valsartan	65.67	
		Candesartan	58.97	
		Irbesartan	49.31	
ETBR	Antagonists	Bosentan	58.26	67.27
		IRL2500	75.09	
		K8794	68.45	
		The total average docking scores	51.68	
